# Development of the annual incidence rate of fracture in children 1980–2018: a population-based study of 32,375 fractures

**DOI:** 10.1080/17453674.2020.1772555

**Published:** 2020-06-05

**Authors:** Andreas V Larsen, Esben Mundbjerg, Jens M Lauritsen, Christian Faergemann

**Affiliations:** a Accident Analysis Group, Department of Orthopaedics and Traumatology, Odense University Hospital, Odense;; b Section for Pediatric Orthopaedics, Department of Orthopaedics and Traumatology, Odense University Hospital, Odense, Denmark

## Abstract

Background and purpose — Pediatric fractures are a common cause of morbidity. So far, no larger Danish study has described the development in the incidence rates. Therefore, we describe the development in the incidence rates of pediatric fractures in the time period 1980–2018 and the frequency of the most common type of fractures.

Patients and methods — This is a retrospective register study of all children aged 0–15 years with a fracture treated in the Emergency Department at Odense University Hospital, Denmark, between 1980 and 2018. For all cases, information on age, sex, date of treatment, diagnosis, and treatment was obtained from the patient registration system. Based on official public population counts we estimated age and sex-specific annual incidence rates.

Results — 32,375 fractures were included. In the study period the incidence rate decreased by 12%. The incidence increased until the early 1990s. Thereafter incidence rates decreased until 2004–09, from then onward increasing towards the end of the study period. The highest age-specific incidence rate in boys of 522 per 10,000 person-years was at 13 years of age. In girls the age of the highest incidence rate decreased from 11 years in 1980 to 10 years in 2018. Fracture of the lower end of the forearm, the clavicle, and the lower end of the humerus had the highest single fracture incidence rates.

Interpretation — The incidence rate of pediatric fractures decreased in the study period by 12%. The highest single fracture incidence rates were for fracture of the lower end of the forearm, the clavicle, and the lower end of the humerus. As the first longitudinal Danish study of pediatric fractures this study is a baseline for evaluating future interventions and future studies.

Injuries are one of the leading causes of morbidity in children and are the leading cause of admission to the healthcare system. In 2018 more than 300,000 children and adolescents were treated in Danish Emergency Departments because of injuries (Statistics Denmark 2018).

Previous studies have found that the overall risk of sustaining a fracture during childhood is 10–25% (Sibert et al. 1981, Landin 1983, Cheng and Shen 1993, Landin 1997). Additionally, the lifetime risk of sustaining a fracture is 27% and 42% for girls and boys respectively (Landin 1997). In a study from Hong Kong the lifetime risk of hospitalization due to a pediatric fracture was 7% (Cheng and Shen 1993).

Studies have demonstrated variations in the incidence rate of childhood fractures. In Sweden an increase in the incidence rate of pediatric fractures was found from 1950 to 1979 (Landin 1983, 1997). A similar increase was found in Finland from 1967 to 1983 (Mäyränpää et al. 2011). Conversely, during the 1980s the incidence rate decreased in these countries (Tiderius et al. 1999, Mäyränpää et al. 2011). Since 1993 different trends have been found in incidence rates as an increase was found in Sweden while a continued decrease was found in Finland (Hedström et al. 2010, Mäyränpää et al. 2011).

Only 1 recent study has described the changes in the incidence rates of pediatric fractures (Lempesis et al. 2017). This Swedish study found a decrease in the fracture incidence rate in girls from 1993–1994 to 2005–2006, but not in boys. So far, no other recent longitudinal population-based study of the variation in the incidence rates of pediatric fractures has been published.

We describe the development in the incidence rates of pediatric fractures in the period 1980–2018 and describe the frequency and changes of most common type of fractures.

## Patients and methods

The population base for this study was the Odense Municipality in Denmark from January 1980 to December 2018. Odense Municipality is a well-defined geographical area with a population of 202,663 in 2018, mainly consisting of the city of Odense (Statistics Denmark 2020). The midyear population of children 0–15 years of age has decreased from 36,665 in 1980 to 33,751 in 2018 (Statistics Denmark 2019a and b, 2020).

The cases included are all children living in Odense Municipality aged 0–15 years with a bone fracture treated in the Emergency Department (ED) at Odense University Hospital (OUH) from 1980 to 2018. The ED at OUH is the only ED in the municipality. All registration was done by qualified staff and registered in a similar manner in the entire study period. In Denmark all registered inhabitants have a unique civil registration number (CPR number), which follows each individual for their entire life. The CPR number was used to identify individuals with recurrent contacts due to the same fracture. In case of more than 1 contact for the same fracture only the 1st contact was registered. Trained physicians determined diagnosis according to the ICD system. ICD-8 was used from 1980 to 1993, and from 1994 onward the ICD-10 was used. Due to differences between ICD-8 and ICD-10 only patients with ICD-10 coding were including for describing changes in fracture types to ensure quality of data.

For all cases information on age, sex, date of treatment, diagnosis, and type of treatment was obtained from the patient registration system.

We defined 4 age groups according to psychosocial and physiological steps of children’s development: 0–1 years were classified as infants, 2–5 years as pre-school children, 6–11 years as schoolchildren, and 12–15 years as adolescents.

Fractures were defined as any bone damage including epiphyseal fractures (Salter–Harris types), complete fractures, incomplete fractures (bowing fractures, greenstick fractures, and torus fractures), avulsions, and Tillaux/triplane fractures. ICD-8 coding had a specific diagnosis for clinical fractures defined as growth zone tenderness combined with swelling without radiological verification. In the whole study period “clinical fractures” have been considered Salter–Harris type 1 fractures and treated as such. Those “clinical fractures” represented 14% of fractures 1980–1993 in all age groups and were excluded from the study. The ICD-10 coding has no specific diagnosis for “clinical fractures.” However, since the percentages of clinical fractures remained constant through all years in all age groups in the ICD-8 period 1980–93, we assumed that the percentage of “clinical fractures” remained constant in the entire study period. Therefore, we excluded the same proportion of fractures in the ICD-10 period 1994–2018, thereby excluding clinical fractures.

The data were grouped and analyzed in 5-year groups. For unknown reasons data from 1995 were corrupted in relation to identifying the patients, rendering them untrustworthy, thus they were excluded entirely from the dataset. Due to the change from ICD-8 to ICD-10 in 1994 we defined the following 4- or 5-year groups with ICD8: 1980–1984, 1985–1989, 1990–1993, and the following groups with ICD10: 1994–1999 (1995 excluded), 2000–2004, 2005–2009, 2010–2014, 2015–2018.

### Statistics

Based on population counts we calculated age- and sex-specific annual incidence rates, with the statistics including 95% confidence intervals (CI). Population counts were extracted as population at risk from Statistics Denmark (Statistics Denmark 2019a and b, 2020). The incident rates were estimated as incidence densities in a dynamic cohort allowing subjects to enter and leave the cohort by migration (Rothman et al. 2008). Children with fractures were not excluded from the population at risk. EpiData Analysis V2.2.2.185 (https://www.epidata.dk/) and Stata 10 (StataCorp, College Station, TX, USA) were used in all statistical analyses. P-values < 0.05 were considered significant.

### Ethics, funding, and potential conflicts of interest

This work is a retrospective register study, which is completely based on patient data existing beforehand. The study was approved by the National Data Protection Agency and the Danish Patient Safety Authority. This study recieved no funding. No benefits in any form have been received or will be received from a commercial party related directly or indirectly to the subject of this work. None of the authors have any conflict of interest to declare. 

## Results

During the 38-year study period the ED at OUH had 253,198 admissions of children due to injuries, of whom 32,375 had fractures. The yearly percentage of all contacts in which a fracture was diagnosed ranged from 12% in 1984 to 17% in 1994. There were 30,621 single fracture incidents. In 1,620 incidents the child had 2 fractures and in 134 incidents the child had 3 fractures. 59% were boys and 41% were girls. In the different age groups the distribution was 0–1 years 49% vs. 51%, 2–5 years 56% vs. 44%, 6–11 years 54% vs. 46%, and 12–15 years 66% vs. 34%. The median age was 11 years for both boys and girls.

The overall annual incidence rate of fractures was 255 (CI 252–258) per 10,000 person-years. The incidence rate was 215 (CI 211–219) for girls and 293 (CI 289–297) for boys. When comparing 1980–1984 with 2015–2018 the overall incidence rate decreased from 284 (CI 276–292) to 249 (CI 240–258) per 10,000 person-years, corresponding to a 12% decrease. The highest incidence rate of 296 (CI 287–304) per 10,000 person-years was found in 1985–1989, which was a 4% increase from 1980–1984. From 1985–1989 to 2015–2018 the incidence rate decreased by 14%.

**Figure 1.**
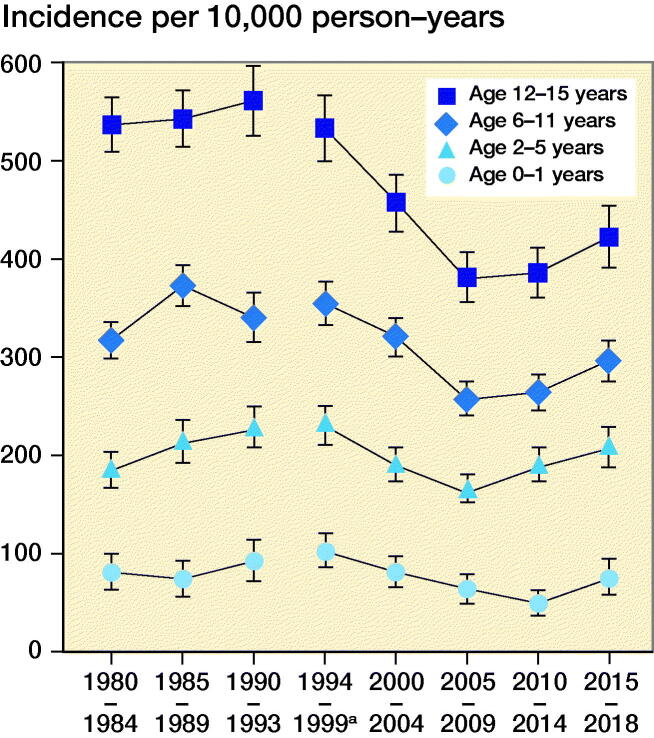
Annual incidence rate of pediatric fractures for boys in different age groups. **^a^**1995 excluded.

**Figure 2.**
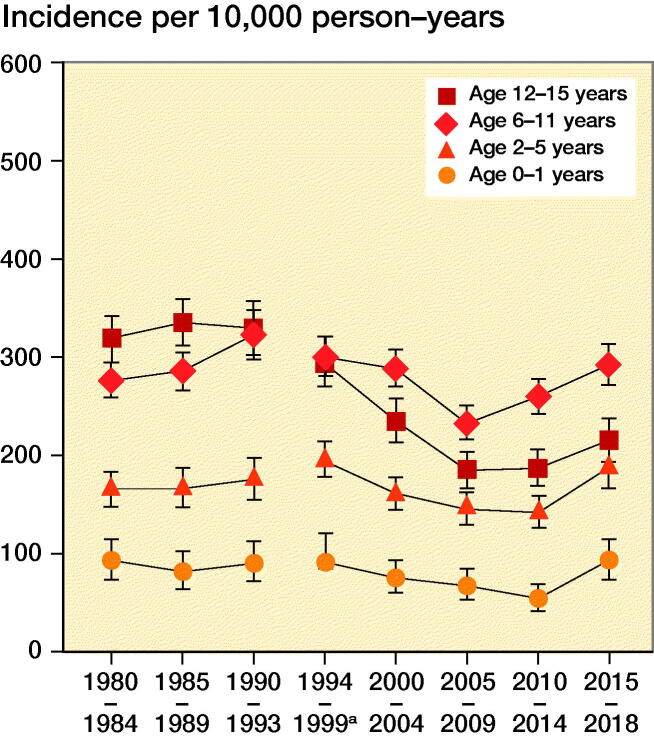
Annual incidence rate of pediatric fractures for girls in different age groups. **^a^**1995 excluded.

There was a statistically significant overall increase in fracture incidence rates during the late 1980s and then decreases during the late 1990s until 2004–2009, whereafter the incidence increases again (Figures 1 and 2).

**Figure 3.**
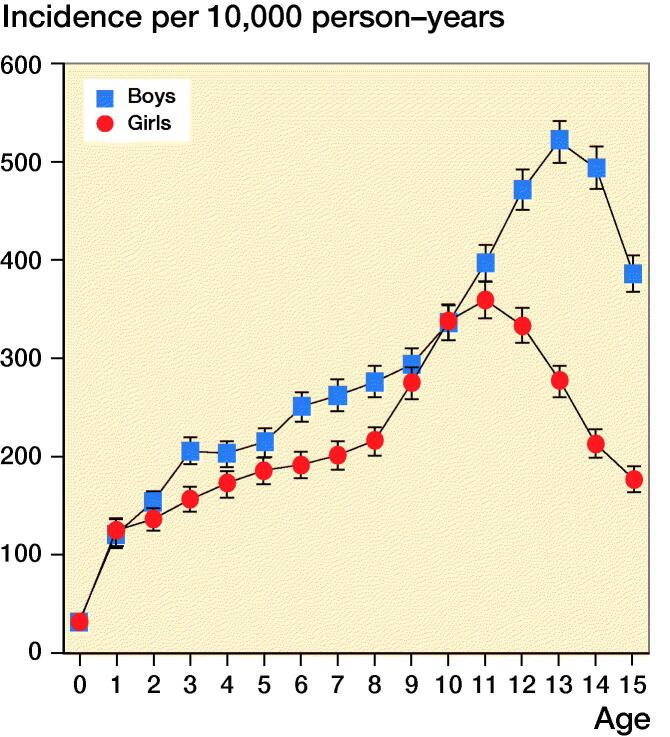
Age-specific annual incidence rates of pediatric fractures stratified by sex.

**Figure 4.**
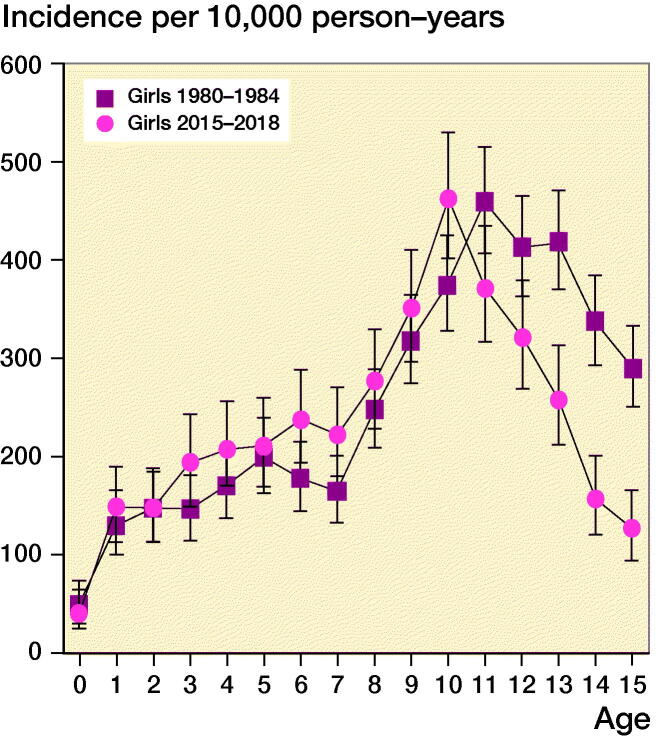
Age-specific annual incidence rate of pediatric fractures in girls in the years 1980–84 and 2010–14.

It should be noted that the decrease in fracture incidence is not evenly spread throughout the different age groups during the study period. The decrease was most pronounced in the age group 12–15 years. This was in fact the only age group with a statistically significant decrease with no overlapping confidence intervals. The decrease was most prominent for girls in this age group for whom the incidence rate decreased by approximately a third from the early 1990s.

In the group of boys, the age specific incidence rates increased until the age of 13 years (Figure 3). Thereafter the incidence decreased with increasing age. In girls, a similar increase was found until the peak at the age of 11 years. After that the incidence decreased.

Figure 4 compares the age-specific annual incidence rate in girls in the years 1980–1984 to 2015–2018. A similar pattern in incidence rates was observed in the 2 time periods. However, in girls the peak incidence was at 11 years in 1980–1984; this changed to 10 years of age in 2015–2018. In boys the age of peak incidence rate remained unchanged in the study period. In girls aged 13–15 we found statistically significantly fewer fractures than during 2015–2018 compared with 1980–1984.

The most common fractures were fracture in the lower end of the forearm involving radius and/or ulna (29%), clavicle (6.4%), the lower end of humerus (4.9%), the metatarsal bone (4.2%), the metacarpal bone (4.1%), finger (3.1%), and the lower end of tibia and/or fibula (2.5%). The overall annual incidence rates per 10,000 population/years were 77 (CI 75–79) for distal forearm fractures, 17 (CI 16–18) for fractures of the clavicle, 13 (CI 12–14) for fractures of the lower end of humerus, 11 (CI 10–12) for fractures of the metatarsal bones, 11 (CI 10–12) for fractures of the metacarpal bones, 8.3 (CI 7.7–9.0) for finger fractures, and 6.7 (CI 6.2–7.3) for fractures of the lower end of tibia and/or fibula.

Fracture of the lower end of radius and/or ulna was the most common fracture in all age groups (Table). A statistically significant decrease was found in the fractures of the metacarpal bones in the age groups 6–11 and 12–15 years.

## Discussion

During the study period the overall annual incidence rate decreased statistically significantly by 12% from 1980–1984 to 2015–2018. The highest incidence rates were found in 1990–1993 or 1994–1999.

Compared with studies from Sweden, we found a higher fracture incidence rate during the 1980s and 1990s (Landin 1997, Tiderius et al. 1999). However, the overall decreasing trend in incidence rates was similar to that in our study. A recent Swedish study found an increasing fracture incidence rate in boys from 1995 to 2006, and a continuously decreasing incidence in girls (Lempesis et al. 2017). A Danish study found similar results in 1988 concerning distal forearm fractures with a higher incidence in Denmark compared with Sweden (Kramhøft and Bødtker 1988).

Contrary to our findings, a Northern Swedish study found an increase in the overall incidence rate of pediatric fractures in the early 2000s (Hedström et al. 2010). In another Swedish study from Malmö the incidence rate of fractures in girls decreased in the early 2000s whereas the incidence rate in boys remained unchanged (Lempesis et al. 2017).

The increase in the incidence rates in the 1980s may be due to changes in leisure activities, as well as more traffic injuries (Landin 1983). The decrease in the incidence rate in our study can be explained by several factors. First, in the late 1980s nurses began visiting homes in Denmark when a child was born in order to secure the surroundings in an attempt to prevent home accidents (Accident Analysis Group 1983). Second, changes in children’s activities and interests during the study period may have led to the decreasing incidence rate. The daily time spend on watching television increased during the 1990s, and computer and internet usage increased during the early 2000s, which may have led to reduced time involving risky activities (Pilgaard 2008, Bille 2008, Lubans et al. 2010). Third, obesity has been suspected to increase the risk of fractures. An Italian study found that obese children have an increased fracture risk at ages 6–12 years. The authors suspect reduced bone density as a result of reduced physical activity, biomechanical factors, and vitamin D deficiency to be the reason for the increased fracture risk (Ferro et al. 2018). However, a Danish study found that the prevalence rates of obesity among children have plateaued and even shown a tendency for decline in the years 1998–2011 (Morgen et al. 2013), a period in which our study found both decrease and increase in fracture incidences. Fourth, children’s fractures have previously been related to socioeconomic status (Hippisley-Cox et al. 2002). The Danish BNP has been steadily increasing over the study period, which may contribute to the decrease. Additionally, safety equipment has been more common over the study period, i.e., wrist and elbow protectors. Notably, comparing the 2004–09 with 2015–18 there was a tendency for increasing incidence. This was statistically significant in both sexes in age groups 2–5 years and 6–11 years as well as in girls in the age group 0–1 years.

The highest incidence rate for boys and girls in our study was 13 and 11 years, respectively. This corresponds to findings of other studies (Landin 1983, 1997, Hedström et al. 2010, Rennie et al. 2007, Meling et al. 2009, Lempesis et al. 2017). We also found that the peak incidence rate in girls decreased from 11 years in 1980–1984 to 10 years in 2015–2018. However, this was not statistically significant. The decrease could correlate with the earlier onset of puberty in Danish girls, thus strengthening the theory that peak fracture incidence in children correlates with maximum growth rate and changes in behavior and interests at onset of puberty (Aksglaede et al. 2009). However, the decrease in fracture incidence was statistically significant for girls aged 13 to 15.

The most common fractures found in this study correspond well to findings in previous studies (Landin 1997, Tiderius et al. 1999, Rennie et al. 2007, Hedström et al. 2010, Mäyränpää et al. 2011). Previous studies found that fracture of the distal radius and/or ulna is the most common fracture, representing 23–33% of all fractures, followed by phalangeal fractures of the hand, clavicle fractures, fractures of the lower end of the humerus, and fractures of the ankle.

The strengths in this study are the long study period with continuous data in a geographically well-defined municipality, the valid population data, and the good registration practice in the ED. There are some limitations to the study design. The annual incidence rates described in our study include only cases requiring medical attention in the Emergency Department at Odense University Hospital. Possible selection bias arises as we have no information available regarding the total number of cases who seek medical attention via general practitioners or in neighboring hospitals 45 km away. However, an earlier study found that very few patients travel for treatment (Lauritsen 1987). Additionally, the number of general practitioners treating fractures is negligible because they do not have access to radiographic equipment. As this was a register-based study, data may be subject to errors in registration. However, all information was registered by the trained staff and diagnosis determined by trained physicians. In 1994 the registration of fractures changed from ICD-8 to ICD-10 coding system. To ensure the quality of the data, only data from 1994 and onward (ICD-10) were used for evaluation of fracture location. However, regarding the total number of fractures during the entire study period both ICD-8 and ICD-10 were used. We have no reason to believe that the change from IDC-8 to ICD-10 in 1994 has produced changes in the overall coding practice of fractures. Any fracture would be classified as such in both ICD-8 and ICD-10 and the coding differs only regarding the anatomical location; thus, the total numbers of fractures should not be influenced.

The chosen method of excluding the estimated proportion of clinical fractures in the ICD-10 period 1994–2018 may lead to a slightly under- or overestimation of the incidence rates in this period. We consider this method necessary to ensure comparability with neighboring countries and other studies. However, in the entire study period tenderness of growth zones combined with swelling without radiographic sign of fracture has been treated and coded as a fracture according to the local guideline for pediatric fracture treatment. We have no reason to believe that practice changed in the study period. Furthermore, there was no systematic change in the incidence rates between the ICD-8 registration in 1990–1993 and the ICD-10 registration in 1994–1999.

This is the first longitudinal Danish study of pediatric fractures. The study provides a baseline for comparing Danish incidence rates with neighboring countries and a baseline for evaluation of future interventions. Furthermore, the study gives important knowledge to emergency departments. However, more detailed studies of injury mechanism in order to prevent pediatric fractures are needed. This study focuses on the total number of fractures among children. However, only a certain percentage of children experience fractures during childhood, and some children experience more than 1 fracture incident. Further studies on the recurrence of fractures in children are needed. Furthermore, studies concerning the decreasing age of puberty among girls correlated to the considerable decrease in fracture incidence are needed.

Incidence rate (IR) per 10,000 population–years of the 4 most common fractures in each age group in the study periods 1994–99 and 2015–18

**Table:** 

Age		1994–1999	2015–2018
Fracture location	ICD10	IR (95% CI)	IR (95% CI)
0–1			
Lower end of radius and/or ulna	DS52.5–DS52.6	26 (20–33)	23 (16–33)
Lower end of tibia	DS82.3–DS82.4	9.8 (6.3–15)	18 (12–25)
Clavicle	DS42.0	14 (6.6–15)	5.6 (2.7–10)
Lower end of humerus	DS42.4	4.9 (2.5–8.6)	5.1 (2.3–9.6)
2–5			
Lower end of radius and/or ulna	DS52.5–DS52.6	54 (48–62)	61 (53–70)
Clavicle	DS42.0	27 (23–33)	24 (19–29)
Lower end of humerus	DS42.4	25 (20–30)	17 (13–22)
Lower end of tibia and/or fibula	DS82.3–DS82.4	9.9 (7.2–13)	11 (7.9–12)
6–11			
Lower end of radius and/or ulna	DS52.5–DS52.6	117 (109–126)	118 (109–128)
Lower end of humerus	DS42.4	16 (13–19)	14 (11–18)
Clavicle	DS42.0	13 (10–16)	12 (9.8–16)
Metacarpal bone	DS62.2–DS62.4	16 (13–19)	6.9 (4.9–9.6)
12–15			
Lower end of radius and/or ulna	DS52.5–DS52.6	85 (77–95)	98 (87–110)
Metacarpal bone	DS62.2–DS62.4	31 (26–36)	17 (13–27)
Clavicle	DS42.0	17 (13–22)	19 (14–24)
Finger	DS62.5–DS62.6	18 (14–22)	12 (7.7–15)
